# Usability Evaluation of eCoaching for Family Carers of Older Adults: Think-Aloud and User Acceptance Testing

**DOI:** 10.1093/geroni/igaf122.034

**Published:** 2025-12-31

**Authors:** Tom Chun Wai Tsoi, Yu Hon Kenneth Kwok, Vera Mun Yu Tang, Wai Sze Chan, Tarani Chandola, Jianchao Quan, Vivian Weiqun Lou

**Affiliations:** The University of Hong Kong, Hong Kong, China (People’s Republic); The University of Hong Kong, Hong Kong, China (People’s Republic); The University of Hong Kong, Hong Kong, China (People’s Republic); The University of Hong Kong, Hong Kong, China (People’s Republic); The University of Hong Kong, Hong Kong, China (People’s Republic); The University of Hong Kong, Hong Kong, China (People’s Republic); The University of Hong Kong, Hong Kong, China (People’s Republic)

## Abstract

Carers of older adults encounter significant barriers in using center-based support services due to caregiving demands and flexed operation hours. Online interventions may help bridge that gap by improving flexibility and accessibility for those who are unable to receive center-based support services. The eCoaching platform promotes mental health and resilience among family carers managing caregiving demands through interactive modules on cognitive behavioral therapy, self-care strategies, resilience-building, lifestyle modifications to enhance carers’ emotional well-being and self-care self-efficacy. Multiple methods were applied in two phases of this formative usability evaluation. In phase one, think-aloud methodology was employed to understand carers’ interactions with the platform and identify usability challenges. Six participants engaged in a 60-minute testing session, verbalizing their thoughts and impressions while navigating the eCoaching platform. Following the think-aloud sessions, semi-structured interviews were conducted to capture retrospective insights regarding participants’ experiences and difficulties encountered. In phase two, usability testing assessed the platform’s functionality over a one-week period. Nine participants used the eCoaching platform for approximately 30 minutes each day. Individual semi-structured interviews gathered in-depth feedback on usability and user experience, covering ease of use, content relevance, accessibility, and visual design. Participants shared their impressions, key takeaways, likelihood of future use, and suggestions for improvement, offering valuable insights for enhancing the eCoaching platform. Results from the usability study with end-users informed refinements to the eCoaching platform before its launch for evaluating its effectiveness in helping family carers of older adults manage their mental health in a three-arm randomized controlled trial in Hong Kong.

